# Oxidative Stress Responses in the Human Fungal Pathogen, *Candida albicans*

**DOI:** 10.3390/biom5010142

**Published:** 2015-02-25

**Authors:** Alessandra da Silva Dantas, Alison Day, Mélanie Ikeh, Iaroslava Kos, Beatrice Achan, Janet Quinn

**Affiliations:** 1 Departamento de Biologia Celular e Genética, Instituto de Biologia Roberto Alcantara Gomes, Universidade do Estado do Rio de Janeiro (UERJ), Rio de Janeiro 20550-013, Brazil; E-Mail: alesdantas@gmail.com; 2 Institute for Cell and Molecular Biosciences, Faculty of Medical Sciences, Newcastle University, Newcastle upon Tyne NE2 4HH, UK; E-Mails: a.m.day@ncl.ac.uk (A.D.); m.ikeh@ncl.ac.uk (M.I.); i.kos@ncl.ac.uk (I.K.); b.achan@ncl.ac.uk (B.A.)

**Keywords:** fungal pathogenesis, *Candida albicans*, oxidative stress, stress signaling

## Abstract

*Candida albicans* is a major fungal pathogen of humans, causing approximately 400,000 life-threatening systemic infections world-wide each year in severely immunocompromised patients. An important fungicidal mechanism employed by innate immune cells involves the generation of toxic reactive oxygen species (ROS), such as superoxide and hydrogen peroxide. Consequently, there is much interest in the strategies employed by *C. albicans* to evade the oxidative killing by macrophages and neutrophils. Our understanding of how *C. albicans* senses and responds to ROS has significantly increased in recent years. Key findings include the observations that hydrogen peroxide triggers the filamentation of this polymorphic fungus and that a superoxide dismutase enzyme with a novel mode of action is expressed at the cell surface of *C. albicans*. Furthermore, recent studies have indicated that combinations of the chemical stresses generated by phagocytes can actively prevent *C. albicans* oxidative stress responses through a mechanism termed the stress pathway interference. In this review, we present an up-date of our current understanding of the role and regulation of oxidative stress responses in this important human fungal pathogen.

## 1. *Candida albicans* Is a Major Fungal Pathogen of Humans

The polymorphic fungus, *Candida albicans*, is a constituent of the normal human microbiome. This fungus, together with other *Candida* family members, is present on the skin and in the oral cavity and gastrointestinal and urogenital tracts of most healthy individuals [1,2]. In the healthy host, *C. albicans* normally exists as a benign commensal organism. However, as an opportunistic pathogen, this fungus can also cause superficial infections, such as oral or vaginal candidiasis, or life-threatening systemic infections [2]. Perturbation of the microbiome through antibiotic usage or mild to severe defects in immune defences, such as in patients with HIV, can result in superficial oral and vaginal infections (thrush), termed oral (OC) and vulvovaginal (VVC) candidiasis, respectively. OC occurs in about 90% of HIV-infected persons as an AIDS-defining illness [3]. Defective immunity in premature infants and the elderly can also result in OC [4,5]. Significantly, 75% of women of childbearing age suffer from VVC, 45% of whom go on to have a least one recurrent infection [6]. Superficial candidiasis can also manifest as chronic infections of the skin and nails, resulting in mucocutaneous candidiasis (CMC) [7]. Although superficial infections are remarkably commonplace, they are non-life threatening and can be easily treated.

In contrast, systemic candidiasis is associated with unacceptably high crude and attributable mortality rates of 42 and 27%, respectively, despite the availability of antifungal drugs, such as the polyenes, azoles and echinocandins. These mortality rates exceed those attributed to sepsis caused by the most aggressive bacterial and viral pathogens [8] and are attributed to difficulties in diagnosing fungal systemic infections and the consequential delays in treatment [9]. Patients who are severely immunocompromised, such as those on immunosuppressive treatments for cancer or transplant surgery, are at risk of systemic candidiasis [10]. In such patients, the innate defence mechanisms, which are vital to prevent invasive disease, are significantly compromised [11]. Consequently, the fungus can survive in the bloodstream and subsequently colonise a number of internal organs [2]. Other risk factors include invasive clinical procedures or trauma, which disrupt the protective anatomical barrier of the mucosa, and the use of venous catheters, which can allow access of the fungus to the bloodstream [10]. Indeed, overall, *Candida* spp*.* are the fourth most common nosocomial (hospital acquired) systemic infection in the United States [8]. Clearly, *C. albicans* poses a significant medical problem, and thus, it is important that we understand what makes this fungus such a successful pathogen.

## 2. Reactive Oxygen Species Are a Core Component of the Immune Cell Armoury

In healthy hosts, the first line of defence against *C. albicans* is through phagocytosis by innate immune cells, including macrophages and neutrophils. A major antimicrobial defence mechanism mounted by these phagocytes is the production of reactive oxygen species (ROS) through a process known as the respiratory burst. Following stimulation by cytokines, phagocytic cells activate the assembly of the NADPH oxidase complex, which results in the generation of superoxide (O_2_^−^•). Given the potency of the ROS produced by NADPH oxidase [12], activation of this multi-subunit enzyme is tightly regulated. The NADPH oxidase complex consists of the Nox2 (gp91^phox^) catalytic subunit, the p22^phox^ transmembrane protein and three cytosolic subunits, p47^phox^, p67^phox^ and p40^ph^°^x^. Nox2 and p22^phox^ make up the membrane-associated cytochrome *b*_558_ heterodimer. Activation of Nox2 is dependent on the interaction with the cytosolic components, in particular p67^phox^, which translocate to the membrane following phagocytosis [13]. This interaction is dependent on the binding of the small GTPase Rac to p67^phox^, which induces a conformation change in this subunit, thus promoting its interaction with Nox2 [14]. Activation of Nox2 drives the production of superoxide via the NADPH-driven reduction of molecular oxygen. This is generated at an extremely high rate of 5 to 10 nmol per s within the neutrophil phagosome [15], and it has been estimated that approximately 4 mol L^−1^ of O_2_^−^• is produced per bacterium engulfed in the phagocytic vacuole [16]. The superoxide is then dismutated to hydrogen peroxide (H_2_O_2_) by superoxide dismutase or to hydroxyl anions (OH^−^) and hydroxyl radicals (OH) via the Haber-Weiss reaction. The importance of the NADPH oxidase-mediated respiratory burst as an antimicrobial mechanism is manifested in patients with chronic granulomatous disease (CGD). CGD is a human genetic disorder characterized by a deficiency in the NAPDH oxidase complex and is associated with recurrent and life-threatening bacterial and fungal infections [17]. Significantly, patients with CGD have an increased susceptibility to *Candida* infections [18]. Interestingly, in addition to the fungicidal roles of ROS, recent work has revealed that the ROS produced by NADPH oxidase also functions to recruit phagocytes to *C. albicans* infection foci. This NADPH oxidase-regulated recruitment of phagocytes is important for efficient phagocytosis, containment of the fungus within the phagocyte and survival of the host [19].

Other toxic chemicals are subsequently derived from the ROS in the phagosome [20]. For example, H_2_O_2_ can react with chloride ions (Cl^−^) to form hypochlorous acid (HOCl) in a reaction catalysed by myeloperoxidase (MPO). In addition, the nitric oxide radical generated by the action of the inducible nitric oxide synthase (iNOS) interacts with superoxide to produce the highly toxic peroxynitrite (ONOO) [21]. Recently, work has also revealed that the combination of reactive oxygen species together with the cationic stress generated during phagocyte maturation underlies the potency of phagocytes in *C. albicans* killing [22]. Thus, phagocytic cells synthesize an array of toxic chemicals that work in combination to promote fungal killing. It is also noteworthy that, in addition to ROS production within the phagosome, phagocytes secrete ROS into the external milieu [23]. Consistent with this, *C. albicans* cells have been shown to mount an oxidative stress response prior to phagocytosis [24]. Furthermore, *C. albicans* will also come in contact with ROS produced by H_2_O_2_-producing bacteria in the mouth and gut. Several commensal bacteria, for example *Enterococcus faecalis* [25] and *Lactobacillus* species [26], secrete ROS into their surroundings, and this may have an inhibitory effect on the growth of *C. albicans* in host niches, other than the phagosomal environment. Consistent with this, using a *Caenorhabditis elegans* model of polymicrobial infection, *E. faecalis* was shown to reduce the virulence of *C. albicans* [27].

The ROS generated within the phagosome creates a toxic environment that induces oxidative stress in *C. albicans*. Indeed, exogenous ROS can induce programmed cell death in this fungal pathogen [28]. ROS interact with proteins, lipids and nucleic acids [29], causing irreversible damage to the pathogen. DNA damage caused by ROS can result in chemical base changes, structural alterations, single- and double-strand breaks and cross-linkage. Lipid peroxidation occurs by a free radical chain reaction, which culminates in peroxidation events at many fatty acid side chains, leading to the damage of the cell membrane. ROS reactions with proteins can lead to the formation of protein-protein cross-links, oxidation of the peptide backbone and reversible or irreversible oxidation of amino acid side chains. Although this can be deleterious to protein function, as discussed below, several oxidative stress-sensing proteins are activated by the reversible oxidation of cysteine residues.

## 3. Response of *Candida albicans* to ROS

### 3.1. Transcriptional Responses to ROS

A well-characterized response of eukaryotic microbes to ROS is the rapid induction of mRNAs that encode oxidative stress detoxification and repair proteins. Interestingly, *C. albicans* is considerably more resistant to oxidative stress than the benign model yeasts, *Schizosaccharomyces pombe* and *Saccharomyces cerevisiae* [30,31]. However, the basis for this resistance does not appear to be due to differences in transcriptional responses to oxidative stress, as all three fungi appear to induce a similar set of core antioxidant genes following exposure to H_2_O_2_ [32,33]. These include catalase (*CAT1*), glutathione peroxidase (*GPX*) and superoxide dismutase (*SOD*) antioxidant-encoding genes, in addition to genes encoding components of the glutathione/glutaredoxin (*GSH1*, *TTR1*) and thioredoxin (*TSA1*, *TRX1*, *TRR1*) systems, which play critical roles in repairing oxidatively-damaged proteins, protein folding and sulphur metabolism. Such oxidative stress-responsive genes are also induced in *C. albicans* following exposure to macrophages or neutrophils [34,35,36,37,38], illustrating that this pathogen induces the respiratory burst in these phagocytes. The analyses of GFP-reporter fusions, under the control of oxidative stress-responsive promoters, have also revealed that *C. albicans* is exposed to significant levels of ROS prior to phagocytosis [24]. In contrast, however, oxidative stress responses do not appear to be induced once *C. albicans* cells have established systemic kidney infections [34,39,40]. Thus, inducible oxidative stress responses appear vital for *C. albicans* to survive phagocytosis by innate immune cells, but are seemingly less important for the fungus to develop systemic infections. Indeed, whilst a number of genes encoding key antioxidants (such as *CAT1*, *TRX1*, *GRX2*, *SOD1*, *SOD5*) are important for virulence in systemic models of infection [41,42,43,44,45], others (including *TSA1*, *GPX*s) are dispensable [46,47].

### 3.2. Transcriptional Responses to ROS Are Inhibited in the Presence of Cationic Stress

In healthy individuals, *C. albicans* cannot evade the oxidative-killing mechanisms mounted by innate immune cells. Such cells prevent infection by employing a battery of toxic chemicals in addition to ROS. For example, phagocytes expose *C. albicans* to cationic fluxes (K^+^) and acidification, as well as to superoxide anions [16,21,48]. However, as *C. albicans* is resistant to each of these individual stresses *in vitro* [30,31], a key question, therefore, is: what accounts for the potency of innate immune defences? Although host microenvironments are complex and dynamic, our understanding of *C. albicans* stress responses is based on studies of individual stresses. Significantly, however, recent work has revealed that *C. albicans* is exquisitely sensitive to combinations of oxidative and cationic stresses [49], which are encountered following phagocytosis. Cationic stress can be imposed *in vitro* by exposure of the fungus to either NaCl or KCl and in the phagocyte is caused by increased flux of K^+^ into the phagosome [16]. Strikingly, exposure to cationic stress results in the inhibition of *C. albicans* oxidative stress responses. This phenomenon has been termed “stress pathway interference” [22]*.* The combinatorial stress-mediated synergistic killing of *C. albicans* contrasts starkly with the stress cross-protection described in model yeasts, whereby exposure to one stress protects against subsequent exposure to a different stresses [50]. The existence of stress pathway interference was revealed through gene expression analysis in *C. albicans*. Transcript profiling showed that H_2_O_2_-induced gene expression is severely attenuated, and intracellular ROS levels increase dramatically, following combinatorial oxidative and cationic stress. For example, key antioxidant genes, such as *CAT1* encoding catalase and *TRR1* encoding thioredoxin reductase, which are significantly induced following H_2_O_2_ stress, fail to be induced following exposure of cells to H_2_O_2_ in the presence of cationic stress [22]. This cationic stress-mediated inhibition of oxidative stress responses appears to be of physiological relevance, as the high fungicidal activity of human neutrophils is dependent on the combinatorial effects of the oxidative burst and cationic fluxes [22]. However, as discussed above, oxidative stress-responsive genes are induced following co-culture of *C. albicans* with phagocytes [34,35], so how is this reconciled with the combinatorial stress-mediated inactivation of such genes? It has been suggested [22] that the activation of *C. albicans* antioxidant genes during interaction with phagocytes may be due to exposure to extracellular ROS prior to engulfment [24]. Furthermore, as exposure of cells to combinatorial oxidative and cationic stresses prevents the normal activation of oxidative stress-responsive genes, this may explain why *C. albicans* oxidative stress genes are not expressed in certain host niches, such as during systemic infections of the kidney, despite the presence of neutrophil infiltrates [34].

### 3.3. Extracellular Antioxidant Enzymes as a Pathogen-Specific Adaptation Mechanism

As *C. albicans* appears to mount standard transcription responses to oxidative stress, the high level of resistance of this pathogen to ROS could, instead, be related to the evolutionary expansion of the SOD family and the fact that this pathogen expresses SODs and other antioxidant enzymes on the cell surface. *C. albicans* contains six SOD enzymes distributed between different cellular compartments. Sod1–3 are intracellular enzymes, while Sod4–6 are glycosylphosphatidylinositol (GPI)-anchored cell wall-associated enzymes. The Cu-/Zn-containing Sod1 is induced following phagocytosis and is required for *C. albicans* to resist macrophage-mediated killing [43]. The extracellular Sods also have vital roles in the detoxification of superoxide radicals generated by phagocytes; co-culture of macrophages with *C. albicans* cells lacking Sod4 and Sod5 leads to massive extracellular ROS accumulation *in vitro* [23]. Consequently, inactivation of Sod4 and Sod5 results in *C. albicans* cells that are exquisitely more susceptible to phagocyte-mediated killing [23,35]. Interestingly, the expression of Sod4 and Sod5 is dependent on the morphology of *C. albicans*, as Sod4 is expressed in yeast cells, whereas Sod5 is a hyphal-induced gene [44,51]. Sod5 is also induced following phagocytosis by neutrophils independently of hyphae formation [35]. Recently, structural analysis of Sod5 revealed that it represents a novel class of superoxide dismutases that only depends on Cu for activity. Furthermore, it is secreted in its apo-form and can readily capture extracellular copper without the aid of a Cu chaperone, which rapidly induces activity [52]. It is suggested that this novel mode of activation is uniquely adapted to the host environment, as macrophages release copper in an attempt to kill invading microbes through copper toxicity [53]. In addition to specific Sods, two key peroxidase enzymes have also been found at the cell surface of *C. albicans*; the thiol-specific peroxidase Tsa1 [47,54] and the peroxide detoxifying enzyme catalase [54]. Tsa1 and Cat1 were identified as major plasminogen-binding proteins in isolated cell wall protein preparations [54], and the cell wall localization of Tsa1 has also been illustrated using fluorescence microscopy [47]. These extracellular mechanisms for protection against ROS likely reflect an adaptation of this pathogenic fungus to prevent the intracellular accumulation of toxic levels of ROS.

### 3.4. Morphogenesis as an Oxidative Stress Response

Following phagocytosis, *C. albicans* can evade oxidative-killing by macrophages and neutrophils by switching from budding to filamentous cells, which can pierce the phagosomal membrane [37]. Not only does this allow the pathogen to escape, but this also results in the *C. albicans*-mediated killing of the phagocyte [55]. It has recently been demonstrated that the ability of *C. albicans* to mount robust oxidative stress responses is vital for this polymorphic pathogen to filament in the phagosome [46,56]. *C. albicans* mutants that are sensitive to ROS *in vitro* fail to filament once phagocytosed and, thus, are trapped within the macrophage and unable to evade phagocyte-mediated killing. Consistent with the requirement of fungal oxidative stress defences to allow filamentation and macrophage escape, the phagocyte NADPH oxidase is important in inhibiting filamentation *in vivo* [57]. The ROS produced by the NADPH oxidase also function to recruit phagocytes, thereby increasing phagocytosis and inhibiting filamentation [19]. Thus, the outcome of the battle between *C. albicans* and innate immune cells appears dependent on the NADPH oxidase-regulated functions of the phagocyte and the robustness of the fungal oxidative stress responses.

The mechanisms underlying *C. albicans* filamentation following phagocytosis remain to be fully explored. A recent study reported that the ROS-induced induction of arginine biosynthesis genes is important for hyphal formation following phagocytosis of *C. albicans* [58]. Moreover, exposure of *C. albicans* to the ROS H_2_O_2_ triggers the filamentation of this polymorphic fungus *in vitro* [42,59]. A close examination of the morphology of these cells revealed that H_2_O_2_-induced filaments are hyperpolarized buds, which are morphologically distinct from hyphae and pseudohyphae filamentous forms [42]. The hyperpolarised bud is a relatively recently characterized filamentous form of *C. albicans* and is normally associated with either mutations or chemicals that perturb cell cycle progression [60,61]. The observation that H_2_O_2_ stimulates hyperpolarised bud formation provided the first example of a physiologically relevant condition that induces this filamentous form of growth in *C. albicans.* Does exposure of ROS following phagocytosis trigger the formation of hyperpolarised buds allowing this pathogen to pierce the phagocyte membrane and escape? Evidence so far indicates that ROS-stimulated hyperpolarized bud formation may not contribute to *C. albicans* filamentation within the macrophage. For example, ROS-sensitive *C. albicans* mutants that cannot filament within the macrophage [56] can form H_2_O_2_-induced hyperpolarized buds *in vitro* [42]. Nonetheless, ROS-stimulated filamentation of *C. albicans* may be beneficial for survival in other host niches, such as the gut, where *C. albicans* co-exists with ROS-producing bacteria.

## 4. Signalling Pathways that Mediate *C. albicans* Responses to ROS

To date, three signalling pathways have been demonstrated to be directly activated in response to ROS in *C. albicans*. These include the Cap1 transcription factor, the Hog1 stress-activated protein kinase and the Rad53 DNA damage checkpoint kinase. Here, we discuss the role and regulation of these pathways in oxidative stress responses in *C. albicans*. Other signalling pathways not known to be activated by ROS, but which contribute to oxidative stress tolerance in *C. albicans* are also briefly summarized.

### 4.1. The Cap1 ROS-Responsive Transcription Factor

In *C. albicans*, the Cap1 transcription factor is the major regulator of the oxidative stress-induced transcriptome and proteome, both *in vitro* [62,63] and *ex vivo*, following exposure to neutrophils [35]. Cap1 is a bZip transcription factor of the AP-1 family and is closely related to the *S. cerevisiae* Yap1 and *S. pombe* Pap1 proteins, which have well-characterized roles in oxidative stress and multi-drug resistance [64,65]. Similarly, *C. albicans cap1Δ* cells are sensitive to several reactive oxygen species and drugs [33,66,67]. Chromatin immunoprecipitation (CHiP) analysis to determine direct targets of Cap1 identified many key antioxidant genes, including *CTA1* and *TRX1*, and those involved in the response to drugs, such as *MDR1* [68]. Cap1 plays a role in recruiting the Ada2 component of the SAGA/ADA histone acetylase co-activator complex to the promoters of oxidative stress and drug-responsive target genes [69,70]. Cells lacking Ada2 are highly sensitive to ROS, and the oxidative stress-induced transcription of key Cap1 target genes is significantly impaired; therefore, Cap1 recruitment of the SAGA complex appears to be a vital component of the oxidative stress response in *C. albicans*.

#### 4.1.1. Regulation of Cap1

**Figure 1:**
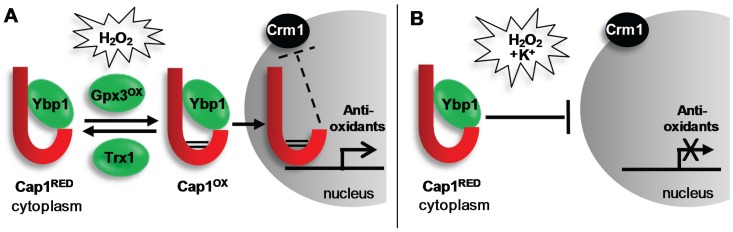
H_2_O_2_-induced activation of Cap1 is inhibited in the presence of cations. (**A**) Exposure of *C. albicans* to H_2_O_2_ promotes the Gpx3/Ybp1-mediated oxidation and activation the Cap1 transcription factor (Cap1^ox^). Cap1^ox^ can no longer interact with the Crm1 nuclear export factor resulting in its nuclear accumulation, and the subsequent Cap1-dependent induction of genes with antioxidant functions necessary for cell survival. Following cellular adaptation, Cap1^ox^ is returned to the inactive reduced form (Cap1^RED^) by thioredoxin (Trx1); (**B**) Remarkably, when *C. albicans* cells are exposed to H_2_O_2_ in the presence of cations, Cap1 fails to accumulate in the nucleus and therefore antioxidant gene expression is not induced leading to cell death. This is important as, following phagocytosis, *C. albicans* is exposed simultaneously to ROS and cationic fluxes. See text for details.

Similar to that reported for *S. cerevisiae* Yap1, *C. albicans* Cap1 rapidly accumulates in the nucleus in response to H_2_O_2_ [56,67]. Under non-stressed conditions, Yap1 shuttles between the cytoplasm and the nucleus due to the interaction of a nuclear export sequence (NES), located at the C-terminus of these transcription factors, with the Crm1 nuclear export factor [71]. However, following exposure to H_2_O_2_, Yap1 is activated by oxidation of specific cysteine residues, resulting in disulphide bond formation between two cysteine-rich domains (n-CRD and c-CRD). This triggers a conformational change within Yap1 that masks the NES, thereby preventing its interaction with Crm1. The inability to be recognized by Crm1 leads to the nuclear accumulation of Yap1, the nuclear-dependent phosphorylation of this transcription factor and the induction of Yap1-dependent genes [72]. Conversely, activation of Yap1 is counteracted by the thioredoxins Trx1 and Trx2, which function to reduce oxidised Yap1 [72]. This basic mechanism of regulation is conserved in *C. albicans* (Figure 1). Mutation of the c-CRD affects Cap1 regulation [67], and Cap1 is rapidly oxidised following exposure to H_2_O_2_ [42]. In addition, following the nuclear accumulation of Cap1, this transcription factor becomes phosphorylated, and the induction of Cap1-dependent genes is observed. Furthermore, as seen in *S. cerevisiae*, thioredoxin functions to reverse the H_2_O_2_-induced oxidation and activation of Cap1 [42].

Fungal AP-1-like transcription factors are not directly oxidised by H_2_O_2_, but instead, specific peroxidase enzymes sense and transduce the H_2_O_2_ signal to these transcription factors (Figure 1). Similar to that observed in *S. cerevisiae* [72], Cap1 oxidation requires Gpx3, a glutathione peroxidase (Gpx)-like enzyme [56]. Studies with Yap1 showed that this transcription factor undergoes multiple oxidation events, with Gpx3 initiating Yap1 oxidation [73,74,75]. Similarly, multiple oxidized forms of Cap1 are also observed [56]. Gpx3-mediated oxidation of Yap1 and Cap1 also requires a second protein, Ybp1, which binds to and forms a complex with the AP-1-like factors [56,76]. A recent study has provided insight into an additional function of Ybp1 in both *C. albicans* and *S. cerevisiae*, as Cap1 and Yap1 are highly unstable in *ybp1Δ* cells [56]. Ubiquitin-mediated degradation of oxidised AP-1-like factors has recently been shown to be an important regulatory mechanism [77,78]; therefore, Ybp1 binding to the reduced cytoplasmic pools of Yap1 or Cap1 possibly functions to prevent this proteasome-mediated degradation [56].

In *S. cerevisiae*, Yap1 functions alongside the Skn7 response regulator transcription factor, to regulate antioxidant gene expression [79]. An orthologue of Skn7 has been identified in *C. albicans* [80]. The overall domain architecture is conserved and comprised of a DNA-binding domain, a coiled-coil domain and a receiver domain (analogous to those in response regulator proteins of two-component signal transduction pathways). It is not known whether Skn7 acts alongside Cap1 in *C. albicans*. However, *C. albicans* cells lacking Skn7 display increased sensitivity to ROS, including H_2_O_2_, consistent with this transcription factor regulating oxidative stress-induced gene expression [80].

Interestingly, Cap1 fails to be activated following exposure to combinatorial oxidative and cationic stress (Figure 1), which underlies the lack of antioxidant gene expression following this combinatorial stress treatment (Section 3.2). In contrast with that seen following oxidative stress, following combinatorial cationic plus oxidative stress treatments, Cap1 fails to accumulate in the nucleus [22]. Consequently, Cap1 is not phosphorylated, and Cap1-dependent oxidative stress genes are not induced [22]. The impact of stress pathway interference upon Cap1 signalling underlies the potency of combinatorial cationic plus oxidative stress, as ectopic expression of the Cap1-dependent catalase gene, *CAT1*, rescues the hypersensitivity to the combinatorial stress [22]. However, the mechanism underlying combinatorial stress-mediated inactivation of Cap1 is not known. Cations inhibit catalase function, which results in high levels of intracellular ROS [22]. Whether high levels of ROS result in Cap1 inactivation or whether cations inhibit Cap1 activation in other ways remains to be determined.

#### 4.1.2. Role of Cap1 in Virulence

Loss of Cap1 or its regulators Gpx3 and Ybp1 attenuates virulence in some, but not all infection models. For example, cells lacking Cap1, Gpx3 or Ybp1 are unable to kill macrophages, due to the inability of these mutant strains to filament following phagocytosis [56]. Consequently, cells lacking Cap1 or its regulators are sensitive to macrophage- and neutrophil-mediated killing [35,56,81]. Cap1, Gpx3 and Ybp1 are also vital for *C. albicans* virulence in a *Galleria mellonella* model of infection [56], and Cap1 is important for virulence in a *Caenorhabditis elegans* infection model in nematode hosts that have a functional NADPH oxidase [81]. In contrast, Cap1, Gpx3 and Ybp1 are dispensable for *C. albicans* virulence in murine systemic infection models [56,81]. Similar findings were reported for Skn7 [80]. The observation that Cap1 is dispensable for virulence in murine systemic models of infection was unexpected, as certain genes that are induced by Cap1 in response to H_2_O_2_, such as *CTA1* and *TRX1*, are important for *C. albicans* survival in such models [42,45]. This indicates that Cap1-independent basal levels of such genes may be important for virulence in such models and that Cap1-mediated gene expression is not vital for the establishment of systemic infections.

### 4.2. The Hog1 SAPK

**Figure 2:**
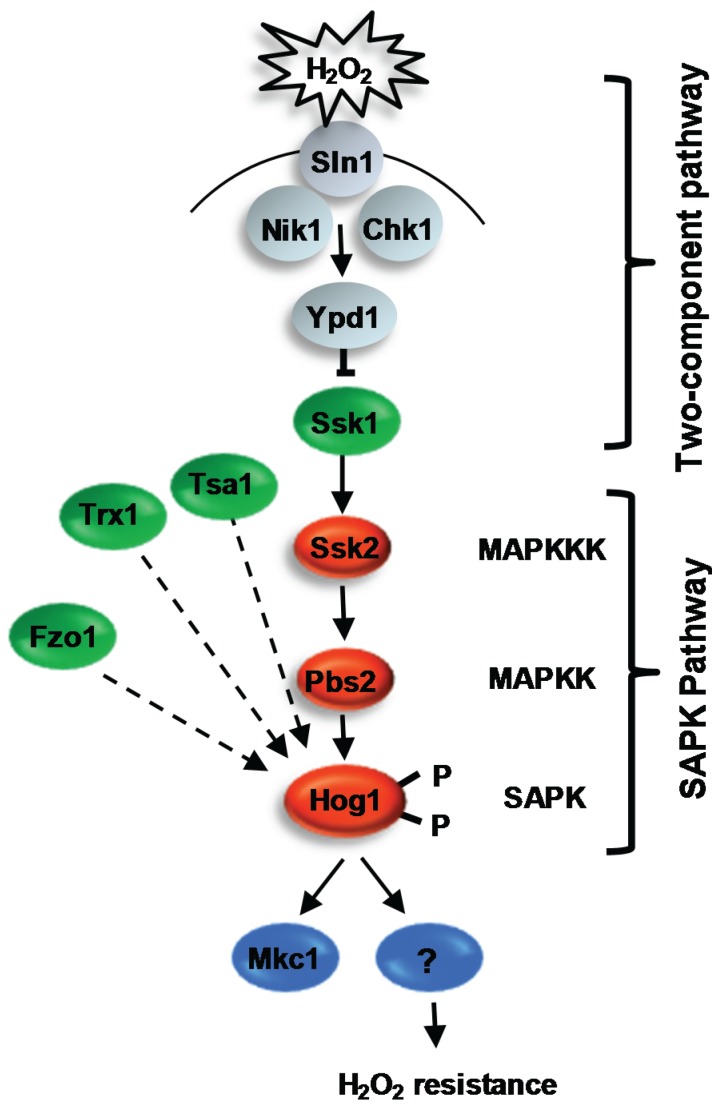
H_2_O_2_-induced activation of the Hog1 SAPK. In response to H_2_O_2_, Hog1 becomes rapidly phosphorylated and accumulates in the nucleus, and *C. albicans* cells lacking Hog1 are sensitive to oxidative stress. Proteins required for H_2_O_2_-induced activation of Hog1 are shown in green. These include the response regulator Ssk1 (but no other two-component protein), the redox sensitive antioxidants Tsa1 and Trx1, and the mitochondria biogenesis factor Fzo1. Following H_2_O_2_-induced activation, Hog1 phosphorylates the Mkc1 MAPK. However, cells lacking Mkc1 are not sensitive to oxidative stress, suggesting that an, as yet, unknown Hog1 substrate(s), mediates oxidative stress resistance.

Stress-activated MAPKs are conserved signalling molecules that promote the ability of cells to adapt to environmental change [82]. They are components of a three-tiered core signalling module that comprises the SAPK itself, a MAP kinase kinase (MAPKK) and a MAPKK kinase (MAPKKK). Activation of the MAPKKK results in the phosphorylation and activation of the MAPKK, which, in turn, culminates in the phosphorylation of the SAPK on conserved threonine and tyrosine residues located within the TGY motif in the phosphorylation lip of the catalytic domain*.* This induces the activation and nuclear accumulation of the kinase [83] and the proline-directed phosphorylation of Ser/Thr residues on diverse substrates, including transcription factors, kinases, cell cycle regulators and membrane proteins, thus eliciting appropriate cellular responses. In *C. albicans*, Hog1 is robustly phosphorylated and rapidly accumulates in the nucleus following exposure of cells to H_2_O_2_ [33]. In addition, cells lacking Hog1 display increased sensitivity to a range of ROS, indicating that Hog1 activation is a critical component of the oxidative stress response in *C. albicans* [84,85]. Interestingly, Hog1 is only activated following exposure of *C. albicans* cells to relatively high levels of H_2_O_2_ compared to the analogous Sty1 SAPK in the model yeast, *S. pombe*. This may reflect an adaption of this pathogenic fungus to restrict Hog1 activation to ROS-rich environments during infection [85]. Despite the increased H_2_O_2_ sensitivity exhibited by *hog1Δ* cells and significant phosphorylation of Hog1 in response to H_2_O_2_, transcript profiling experiments revealed that Hog1 is largely dispensable for H_2_O_2_-induced gene expression [33]. Although a small subset of H_2_O_2_-responsive genes were identified that showed Hog1-dependent induction, subsequent analysis failed to identify any genes coding for proteins with known antioxidant functions [33]. This is in contrast with *S. pombe*, where Sty1 is required for the activation of the core stress genes in response to H_2_O_2_, including genes encoding important antioxidants, such as catalase and glutathione peroxidase [86]. What, therefore, is the role of Hog1 in the *C. albicans* oxidative stress response if it is not required for the induction of antioxidant gene expression? One possibility is that Hog1 contributes to the oxidative stress response at a post-transcriptional level in *C. albicans*. Indeed, the *S. pombe* Sty1 SAPK has been shown to interact with translation factors [87]. However, Hog1 does not play a major role in regulating the oxidative stress-induced proteome, although proteomic experiments did indicate that Hog1 might be required to ensure the prolonged expression of some proteins during recovery from H_2_O_2_ stress [88]. Loss of Hog1 has been shown to affect respiratory function [89], although it is unclear whether this underlies the sensitivity of *hog1Δ* cells to ROS. One downstream target of Hog1 regulated by H_2_O_2_ stress is the Mkc1 cell integrity MAPK. Mkc1 is rapidly phosphorylated in response to H_2_O_2_ stress in a Hog1-dependent mechanism, although Mkc1 is not required for cell survival in response to H_2_O_2_ stress [90]. In addition, the Sko1 transcription factor is a target of the Hog1 SAPK in *C. albicans*, as this becomes phosphorylated following stress in a Hog1-dependent manner [91]. However, consistent with Hog1 not playing a major role in regulating oxidative stress-induced gene expression, the H_2_O_2_-induced transcriptome is not dependent on Sko1 [92]. Thus, in *C. albicans*, Hog1 regulation of the oxidative stress response must involve targets in addition to Mkc1 and Sko1 (Figure 2).

#### 4.2.1. Regulation of Hog1 in Response to ROS

Whilst little is known regarding the cellular targets of Hog and the cellular role(s) of this kinase in promoting oxidative stress tolerance, more progress has been made in delineating how H_2_O_2_ signals are relayed to Hog1 (Figure 2). Oxidative stress-induced activation of Hog1 is entirely dependent on the Pbs2 MAPKK [93], which, in turn, is regulated by a single MAPKKK Ssk2 [94]. Furthermore, a recent study reported that deletion of a mitochondrial biogenesis factor, Fzo1, significantly impairs the H_2_O_2_-induced activation of Hog1 [95]. Thus, intriguingly, functional mitochondria may play an important role in the regulation of the Hog1 pathway in response to oxidative stress. In addition, both two-component related proteins and redox-sensitive antioxidants are necessary for the activation of the Hog1 SAPK in response to oxidative stress, and these will be described in turn.

#### 4.2.1.1. Two-Component Mediated Regulation of Hog1

In the model yeasts, two-component signalling pathways have been shown to play an important role in the sensing and transmission of stress signals to their respective SAPK pathways. Such pathways are comprised of a histidine kinase, an intermediary phosphorelay protein and a response regulator protein. In *S. cerevisiae*, the Sln1 histidine kinase is inactivated in response to osmotic stress. This halts phosphorelay through the Ypd1 phosphorelay protein, leading to the rapid dephosphorylation of the Ssk1 response regulator. Dephosphorylated Ssk1 is a potent activator of the Ssk2/Ssk22 MAPKKKs in *S. cerevisiae*, which regulate Hog1 activation [96,97]. In *C. albicans*, deletion of the analogous *SSK1* gene prevents Hog1 activation in response to oxidative stress, and consistent with this, *ssk1Δ* cells are sensitive to oxidative stress [98]. Although Ssk1 is involved in the transmission of oxidative stress signals to Hog1, the identity of the histidine kinase(s) responsible for sensing and signalling oxidative stress signals to Ssk1 in *C. albicans* remains elusive [99,100]. Of the three histidine kinases present in *C. albicans*, Chk1 would appear to be the most likely candidate for a potential peroxide-sensing histidine kinase, as this shows significant similarity to the *S. pombe* peroxide-sensing histidine kinases, Mak2 and Mak3 [101,102]. However, deletion of *CHK1* alone or in combination with either of the genes encoding the two remaining histidine kinases, *SLN1* or *NIK1*, does not impair H_2_O_2_-induced activation of Hog1 [100,103]. Hence, it is currently unclear as to which histidine kinase(s) senses oxidative stress and regulates phosphorelay to Ssk1. Moreover, observations that Hog1 activation is seen in cells expressing a non-phosphorylatable Ssk1 mutant [103] or in cells lacking the Ypd1 phosphorelay protein in which Ssk1 is predicted to be unphosphorylated [104] indicate that Ssk1 may relay H_2_O_2_ signals to Hog1 in a mechanism independent of two-component signalling. It is also noteworthy that a novel response regulator, named Crr1/Srr1, has been recently identified that is only present in fungi belonging to the *Candida* clade [105,106]. Cells lacking Crr1 or expressing a mutant lacking the predicted aspartate phosphorylation site are sensitive to H_2_O_2_ [105]. However, in contrast with Ssk1, Crr1 is not required for the H_2_O_2_-induced activation of Hog1 [105]. Thus, whilst this novel response regulator mediates the response of *C. albicans* to H_2_O_2_, it does so in a Hog1-independent manner. Finally, in *S. cerevisiae*, the transmembrane protein, Sho1, relays osmotic stress signals to the Hog1 SAPK in parallel with the Sln1-mediated two-component signalling pathway [107]. In *C. albicans*, the analogous Sho1 protein appears to have been reassigned to oxidative stress signalling [100]. However, it is not clear how this is mediated, as Sho1 is not required for ROS-stimulated activation of the Hog1 pathway [100].

#### 4.2.1.2. Redox-Sensitive Antioxidant Proteins as Regulators of Hog1

It is now well recognized that redox-sensitive antioxidant proteins have important sensing and signalling roles in the cellular response to oxidative stress [108]. In *C. albicans*, the redox-sensitive thioredoxin peroxidase enzyme, Tsa1, is specifically required for H_2_O_2_-induced activation of Hog1 [42]. This is similar to that previously reported in *S. pombe*, as H_2_O_2_-induced activation of the Sty1 SAPK also requires the analogous thioredoxin peroxidase enzyme, Tpx1 [109]. In *S. pombe*, intermolecular disulphide bonds are formed between conserved cysteine residues in Sty1 and Tpx1 following H_2_O_2_ stress, which suggests that Tpx1 regulates Sty1 function directly. However, the mechanism of Tsa1 regulation of Hog1 in *C. albicans* may be different, as the conserved peroxidatic cysteine residue of Tsa1, which is essential for Tpx1 regulation of Sty1, is dispensable for Tsa1 regulation of Hog1 [42]. Furthermore, the thioredoxin enzyme, Trx1, which regulates the redox status of Tsa1, is also essential for the relay of oxidative stress signals to the Hog1 SAPK module [42]. eletion of *TRX1* or mutation of the catalytic cysteine residues of Trx1 drastically impairs Hog1 phosphorylation in response to H_2_O_2_. However, it would appear that Trx1 regulates Hog1 independently of Tsa1, as the catalytic cysteine residues of Tsa1, which are reduced by Trx1, are dispensable for Hog1 activation [42]. In mammalian systems, thioredoxin functions as a repressor of the Hog1-related JNK and p38 SAPK signalling cascades [110]. The upstream Ask1 MAPKKK in the mammalian SAPK pathways is activated via cysteine oxidation, and Trx1 negatively regulates this pathway by reducing the oxidized cysteines of Ask1 [111,112]. As Trx1 is a positive regulator of the Hog1 SAPK in *C*. *albicans*, it seems unlikely that a similar mechanism is in place. It is also interesting to note that protein tyrosine phosphatases, which are negative regulators of SAPKs, are susceptible to inactivation by oxidation of their catalytic cysteine residue [113]. Whether thioredoxin regulates such phosphatases that dephosphorylate Hog1 in *C. albicans*, however, remains to be established.

#### 4.2.2. Role of the Hog1 SAPK in Virulence

The stress-activated MAPK Hog1 in *C. albicans* is phosphorylated and accumulates in the nucleus, in response to a range of stresses likely to be encountered in the host, including ROS, osmotic stress and anti-microbial peptides [114]. Cells lacking Hog1 display impaired virulence in a wide range of infection models, including murine systemic and commensal models [114,115,116], and are more susceptible to killing by macrophages or neutrophils [117]. As Hog1 regulates a number of distinct stress responses, it is difficult to dissect whether it is the role of Hog1 in oxidative stress responses or a different aspect of Hog1 signalling that is important for virulence in these models. Importantly, however, although Hog1 signalling has also been implicated in morphogenetic regulation, mutational analysis has inferred that the importance of Hog1 in virulence is due to its role in stress protection, rather than its role in repressing the yeast to hyphal transition [115].

### 4.3. The Rad53 DNA Damage Checkpoint Kinase

**Figure 3:**
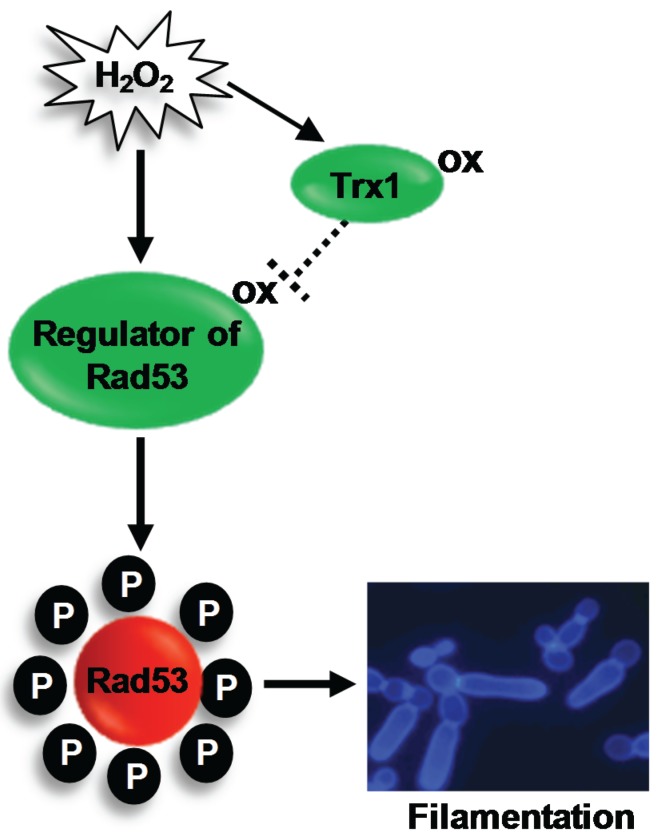
H_2_O_2_-induced activation of Rad53 triggers filamentation in *C. albicans*. The redox sensitive oxidoreductase Trx1 inhibits H_2_O_2_-induced activation of the DNA damage checkpoint kinase Rad53. This suggests that a regulator of Rad53 is activated by oxidation, and this active oxidised form is reduced by Trx1. Activation of the DNA damage checkpoint triggers the formation of hyperpolarised buds. See text for details.

Following exposure to H_2_O_2_, *C. albicans* forms hyperpolarised buds, which are morphologically distinct from hyphae and pseudohyphae filamentous forms (Section 3.4). Consistent with this, H_2_O_2_-induced hyperpolarized bud formation occurs independently of the key hyphal regulators, Efg1 and Cph1, and, instead, depends on the activation of the Rad53 DNA damage checkpoint pathway [42,61] (Figure 3). A wide range of genotoxic stresses, including UV, methyl methanesulfonate (MMS) and the ribonucleotide reductase inhibitor hydroxyurea have been shown to activate the Rad53 kinase in *C. albicans* [61], and loss of Rad53 or upstream regulators of Rad53, prevents hyperpolarised bud formation [61,118]. ROS are also genotoxic agents due to the induction of DNA damage [119], which, in turn, triggers the activation of the Rad53 DNA checkpoint pathway [120]. Indeed, treatment of *C. albicans* cells with H_2_O_2_ elicits the phosphorylation of Rad53, and cells lacking *RAD53* fail to form hyperpolarised buds in response to H_2_O_2_ [42].

#### Regulation of Rad53

Regarding the H_2_O_2_-mediated activation of Rad53, a recent study illustrated that H_2_O_2_-induced oxidation, and inactivation, of the thioredoxin protein Trx1 is important for the activation of Rad53 and polarized cell growth [42]. Rad53 is constitutively phosphorylated in cells lacking Trx1, which display a hyperpolarized bud morphology. Conversely, ectopic expression of the thioredoxin reductase gene, *TRR1*, which reduces oxidized Trx1, inhibited H_2_O_2_-induced filamentation [42]. Taken together, these results illustrate that oxidation of Trx1 following H_2_O_2_ exposure is key in the activation of Rad53 that drives hyperpolarised bud formation. The finding that Trx1 inhibits Rad53 activation under non-stressed conditions may be conserved in higher eukaryotes, as ectopic expression of thioredoxin inhibits the phosphorylation of the analogous DNA damage checkpoint kinase, Chk2, in mammalian cells [121]. However, the mechanism of Trx1 regulation of Rad53 is unclear. As Trx1 functions to reduce oxidised proteins, an attractive hypothesis is that Rad53, or a regulator of this kinase, is activated by oxidation (Figure 3). In this regard, it is interesting that the human homologue (ATM) of the fungal Tel1 DNA-damage sensing kinase, which regulates Rad53, has recently been shown to be activated by oxidation [122]. Further studies are needed to determine if Tel1 is similarly regulated to mediate H_2_O_2_-induced filamentation in *C. albicans*.

### 4.4. Other Signaling Pathways that Contribute to Oxidative Stress Resistance

The cAMP/PKA signalling pathway has a negative impact on oxidative stress responses in *C. albicans*. For example, induction of the pathway by inactivation of the phosphodiesterase, Pde2, which degrades cAMP, results in increased sensitivity to H_2_O_2_ [123]. Related to this, farnesol treatment of *C. albicans* cells results in increased resistance to H_2_O_2_, due to the inhibition of the cAMP/PKA signalling pathway [124]. Such changes in resistance are possibly due to changes in the levels of anti-oxidant gene expression [124]; however, the mechanism linking cAMP/PKA to their regulation is unknown.

There is also evidence that the spindle assembly checkpoint is required for *C. albicans* oxidative stress resistance. Cells lacking the spindle checkpoint protein kinase, Mps1, are sensitive to H_2_O_2_ [125] and, similar to other oxidative stress sensitive mutants [56], fail to filament following phagocytosis. Related to this, the spindle assembly checkpoint protein, Mad2, is essential for *C. albicans* survival in macrophages [126].

## 5. Conclusions and Future Perspectives

In this review, we have summarized the current literature of oxidative stress responses and how they are regulated in the human fungal pathogen *C. albicans*. This is an important area of research, as oxidative stress adaptation is emerging as an important virulence trait in this, and other, fungal pathogens. Table 1 summarizes studies that have documented the impact of the loss of oxidative stress regulatory proteins or antioxidant enzymes on *C. albicans* virulence in either a murine systemic infection model or a macrophage/neutrophil phagocyte-survival infection model. From this summary, a number of observations can be made. First of all, when examined, mutants that display an impaired tolerance to oxidative stress show an impaired ability to survive phagocyte killing. This is consistent with previous observations that *C. albicans* mounts a robust transcriptional response to oxidative stress following phagocytosis [34,35,36,37,38]. Secondly, not all oxidative stress-sensitive *C. albicans* mutants display attenuated virulence in a murine systemic infection model. Perhaps most striking is the observation that the major regulator of anti-oxidant gene expression, Cap1, is dispensable for virulence in such an infection model. This is particularly intriguing, as certain genes, which are dependent on Cap1 for induction following oxidative stress, are important for virulence in systemic models. Thirdly, the role of many other oxidative stress-responsive proteins in mediating *C. albicans* virulence, such as the Rad53-mediated DNA damage checkpoint pathway, have yet to be explored.

**Table 1:** Summary of the role of oxidative stress-responsive signalling proteins and antioxidant enzymes in *C. albicans* virulence. The importance of proteins needed for resistance to oxidative stress in mediating *C. albicans* virulence in either a systemic infection model (SIM) or phagocyte infection model (PIM) is indicated; +, important for virulence; −, dispensable for virulence; nd, not determined. For further explanation, see the text.

Protein	Function	SIM	PIM	References
*Signalling Proteins*				
Hog1	Stress-activated protein kinase	+	+	[114,115,117]
Ssk1	Response regulator	+	+	[127,128]
Cap1	Transcription factor	−	+	[56,81]
Ybp1	Cap1 regulator	−	+	[56]
Gpx3	Cap1 regulator	−	+	[56]
Skn7	Transcription factor	−	nd	[80]
*Signalling Proteins*				
Pde2	Phosphodiesterase	+	nd	[123]
Mps1	Spindle checkpoint	nd	+	[125]
Mad2	Spindle checkpoint	+	+	[126]
*Antioxidant Enzymes*				
Cat1	Catalase	+	+	[45]
Trx1	Thioredoxin	+	nd	[42]
Tsa1	Thioredoxin peroxidase	−	nd	[47]
Sod1	Superoxide dismutase	+	+	[43]
Sod5	Superoxide dismutase	+	+	[23,44]
Grx2	Glutaredoxin	+	nd	[41]
Gpx31-33	Glutathione peroxidases	nd	+	[46]

In addition to gaps in our knowledge regarding the relative importance of specific oxidative stress responses in mediating *C. albicans* virulence, there are also additional key questions that remain to be addressed. For example, what is the role of the Hog1 SAPK in mediating oxidative stress resistance in *C. albicans*, and how is this regulated? This is important, as, although Hog1 is an essential virulence determinant in *C. albicans*, the conservation with highly related SAPKs in human cells suggests that Hog1 itself may be unsuitable as a specific antifungal target. Thus, there is much interest in identifying fungal-specific SAPK regulators or substrates, as such proteins hold greater promise for future therapeutic strategies. In addition, the recent findings that *C. albicans* is exquisitely sensitive to combinations of stress that are encountered following phagocytosis represent a new unchartered area in the field of stress signalling. A key question for the future is how do combinations of stress imposed by the phagosome inhibit oxidative stress adaptation and survival of *C. albicans*? Addressing this question is critical to further our understanding of *Candida*-host interactions during disease progression.
